# Localized and Systemic Immune Responses against SARS-CoV-2 Following Mucosal Immunization

**DOI:** 10.3390/vaccines9020132

**Published:** 2021-02-06

**Authors:** Shaswath S. Chandrasekar, Yashdeep Phanse, Rachel E. Hildebrand, Mostafa Hanafy, Chia-Wei Wu, Chungyi H. Hansen, Jorge E. Osorio, M. Suresh, Adel M. Talaat

**Affiliations:** 1 Department of Pathobiological Sciences, School of Veterinary Medicine, University of Wisconsin, Madison, WI 53706, USA; schandrasek5@wisc.edu (S.S.C.); rhildebrand@wisc.edu (R.E.H.); hanafy@wisc.edu (M.H.); chiaweiwu@wisc.edu (C.-W.W.); chungyi.hansen@wisc.edu (C.H.H.); jorge.osorio@wisc.edu (J.E.O.); suresh.marulasiddappa@wisc.edu (M.S.); 2 Pan Genome Systems, Madison, WI 53719, USA; phanse@pangenosys.com; 3 Department of Microbiology and Immunology, Faculty of Veterinary Medicine, Cairo University, Giza 12211, Egypt; 4 Colombia Wisconsin One Health Consortium, Universidad Nacional Medellín, Calle 75#79a 5, Colombia

**Keywords:** SARS-CoV-2, COVID-19, intranasal vaccine, nanovaccine, heterologous vaccine

## Abstract

The rapid transmission of SARS-CoV-2 in the USA and worldwide necessitates the development of multiple vaccines to combat the COVID-19 global pandemic. Previously, we showed that a particulate adjuvant system, quil-A-loaded chitosan (QAC) nanoparticles, can elicit robust immunity combined with plasmid vaccines when used against avian coronavirus. Here, we report on the immune responses elicited by mucosal homologous plasmid and a heterologous immunization strategy using a plasmid vaccine and a Modified Vaccinia Ankara (MVA) expressing SARS-CoV-2 spike (S) and nucleocapsid (N) antigens. Only the heterologous intranasal immunization strategy elicited neutralizing antibodies against SARS-CoV-2 in serum and bronchoalveolar lavage of mice, suggesting a protective vaccine. The same prime/boost strategy led to the induction of type 1 and type 17 T-cell responses and polyfunctional T-cells expressing multiple type 1 cytokines (e.g., IFN-γ, TNFα, IL-2) in the lungs and spleens of vaccinated mice. In contrast, the plasmid homologous vaccine strategy led to the induction of local mono and polyfunctional T-cells secreting IFN-γ. Outcomes of this study support the potential of QAC-nano vaccines to elicit significant mucosal immune responses against respiratory coronaviruses.

## 1. Introduction

COVID-19 represents a significant challenge to public health authorities worldwide because of the speed of disease transmission and the lack of effective treatment or prevention strategies [1]. The development of traditional vaccines, such as inactivated or live-attenuated vaccines, is time-consuming and may not meet the need for rapid vaccine development. For the inactivated vaccines, there are safety concerns such as incomplete inactivation of the pathogen and the need to grow large volumes of deadly pathogens [2]. On the other hand, live-attenuated vaccines have been shown to induce an antibody-dependent enhancement effect worsening the clinical outcome of rhesus macaques infected with SARS-CoV [3]. Other vaccination strategies such as subunit, RNA-based, and viral vector vaccines have advantages over traditional vaccines; however, their use faces many challenges. For example, subunit vaccines are expensive to produce with limited global production capacity and typically have low immunogenicity [4]. Amidst a global pandemic, there is a need for rapid development of vaccines. Vectored (DNA and viral) vaccines are advantageous because plasmids encoding antigens can be developed within a few days with current rapid and inexpensive gene synthesis technologies [5]. For viral vectored vaccines like the Modified Vaccinia Ankara (MVA) strain, highly efficient scale-up production processes have been set-up already, enabling its utility during a pandemic such as COVID-19 [6].

Previously, experimental plasmid vaccines have been developed for emerging infections, including SARS-CoV, SARS-CoV-2, MERS coronavirus (CoV), Influenza A, and Zika virus [7]. DNA-based vaccines offer several advantages over the other technologies discussed above, including rapid vaccine production and scale up within a few days, once needed (i.e., on-demand manufacturing) [5]. Furthermore, multiple vaccine constructs can be used together [8] to immunize patients effectively, offering highly flexible vaccination programs. Importantly, synthetic DNA is temperature stable and does not need the cold chain needed for live-attenuated or subunit vaccines, thus lowering the cost of vaccine administration and making the supply logistics easier in resource-limited countries. A major challenge with DNA vaccines, however, is the in vivo degradation of the construct by DNases, inefficient uptake by antigen-presenting cells (APC), and low immunogenicity [9,10]. Fortunately, articulate delivery systems such as quil-A-loaded chitosan (QAC), represent a significant improvement over other traditional vaccines by prolonged release of active plasmid expressing antigens as shown before [11]. QAC particulate adjuvant system is composed of chitosan, a biodegradable natural polysaccharide that readily complexes with DNA owing to its positive charge and quil-A, a potent adjuvant with mild surfactant properties [12,13]. The QAC adjuvant system can both mediate direct delivery of plasmid DNA to target cells and act as an antigen depot, releasing DNA payload over time [11]. QAC-adjuvanted vaccines appear to target local mucosal immunity where airway epithelium T-cells and IgA humoral responses have been shown to be critical for restricting respiratory viral pathogens like SARS- and MERS-CoV [14].

Although the correlates of protection against a COVID-19 are still ambiguous, most vaccine efforts are focused on generating neutralizing antibodies, and not much attention has been given to CD4+ and CD8+ T-cell responses [15,16,17,18]. Patients recovered from SARS infection had undetectable anti-SARS antibodies and memory B cells; however, the CD8+ T-cells persisted many years after the infection [19,20]. Furthermore, a recent study showed that the antibody level in many SARS-CoV-2 recovered patients declined to baseline levels within three months, clearly highlighting that the vaccines relying solely on a neutralizing antibody response may not confer long-term protection against SARS-CoV-2 and other coronaviruses [21]. Thus, vaccines against SARS-CoV-2 that can induce both humoral and cellular immune response may have a more durable protective immune response than vaccines just focused on neutralizing antibodies. Such a strategy has been investigated in this report.

Currently, most experimental DNA vaccines are only amenable for intramuscular administration limiting mucosal immunity critically needed to reduce viral infection. Furthermore, the recently approved nucleic acid vaccines for COVID-19 are also administered parenterally [15]. Previously, we had shown that a 2-dose QAC encapsulated plasmid DNA (pQAC) encoding the nucleocapsid gene against avian coronavirus provided equal protection to live-attenuated vaccines when birds were challenged with Infectious Bronchitis Virus [11]. Although comparable, only T-cell responses were observed without a complementing humoral response in birds vaccinated with pQAC. In this report, we hypothesized that a heterologous strategy with QAC encapsulated plasmid DNA (pQAC) prime, followed by an MVA boost, would elicit a broader immune response. Both the pQAC plasmids (pQAC-CoV) and MVA vector (MVA-CoV) were designed to express S and N antigens encoded by SARS-CoV-2 from the early phase of the COVID-19 pandemic. The prime/boost (P/B) of vector vaccines were delivered via the intranasal route (I.N) and hereafter referred to as either pQAC/MVA-CoV or heterologous vaccine. Our results indicated that the I.N immunization with pQAC/MVA-CoV-induced systemic and local neutralizing antibodies in mice. Humoral responses were also complemented by the induction of localized Th17 cellular responses. Moreover, mice vaccinated with only plasmid vectors (pQAC-CoV) via the I.N or intramuscular route (I.M) generated significant type 1 and type 17 (Tc17 or Th17) cellular responses.

## 2. Materials and Methods

### 2.1. Cell Lines

HEK 293T and Vero E6 cells were a kind gift from Dr. Jorge Osorio. Chicken embryonic fibroblasts (CEF) were prepared from 9-day-old-specific pathogen-free (SPF) white leghorn eggs (Charles River Avian Vaccine Services, Norwich, CT, USA) as described previously [22]. Human embryonic kidney cells (HEK-293T) expressing human angiotensin-converting enzyme 2, HEK-293T-hACE2 cell line, NR-52511 was obtained through BEI Resources, NIAID, NIH. All cells were maintained in DMEM supplemented with 10% fetal bovine serum (FBS) and penicillin-streptomycin (D10) at 37 °C, 5% CO_2_ atmosphere.

### 2.2. Preparation of SARS-CoV-2 Vaccine Constructs

Sequences for the SARS-CoV-2 spike (S) and nucleocapsid (N) were downloaded (GenBank accession number MN908947), back-translated, and codon-optimized for expression in mice. DNA fragments encoding truncated S (TrS) with deleted trans-membrane domain and N antigens were commercially synthesized and cloned into a pCMV backbone expression vector C-terminus 6XHis tag (Twist Bioscience, San Francisco, CA, USA). To confirm the insertion of genes in the correct orientation, DNA sequencing was performed at the UW-Madison Biotechnology Center with an ABI Prism 3730XL DNA analyzer using BigDye terminators (Applied Biosystems, San Francisco, CA, USA). To confirm the expression of TrS and N proteins, HEK 293T cells seeded in 6-well format were transfected with an optimized ratio of DNA (3 μg):FuGENE HD (9 μL) in accordance with the manufacturer’s instructions (Promega, Madison, WI, USA). Three days post-transfection, cells, and supernatant (separately) were harvested for western blot analysis. The MVA expressing N and TrS constructs were generated as described before in CEF cells [23]. The cell and supernatant fractions were boiled in Laemmli sample buffer (BioRad, Hercules, CA, USA) and resolved on a 4–20% SDS-PAGE gel by electrophoresis using a Mini-PROTEAN 3 system (BIO-RAD, Irvine, CA, USA). Polyacrylamide gels were electroblotted onto nitrocellulose membranes using a Turboblot^®^ system. Membranes were blocked in 5% (*w*/*v*) skim milk and probed with Direct-Blot™ HRP anti-6-His Epitope Tag Antibody (BioLegend, #906109) or polyclonal mouse anti-SARS-CoV-2 spike sera harvested from one pQAC/MVA-CoV immunized C57BL/6 mouse three weeks post final boost. Membranes were developed using a solid phase 3, 30, 5, 50-tetramethylbenzidine (TMB) substrate system. Plasmid-loaded QAC particles were synthesized as described previously [11].

### 2.3. Vaccine Efficacy Study

The immunogenicity of the experimental vaccine constructs was evaluated in C57BL/6 mice (6 weeks of age) obtained from Taconic Inc. and maintained in bio-safety level-2 containment. At every indicated time point, mice were concurrently immunized with MVA or pQAC TrS and N constructs. A total of 50 mice was divided equally into five groups (*n* = 10 each). Groups of mice were either unvaccinated (PBS) or immunized with pQAC-CoV (I.M) or pQAC-CoV (I.N) at week-0, week-3, and week-6. Another group of C57BL/5 mice was vaccinated with pQAC-CoV (I.N) at week-0, followed by a boost with MVA-CoV (I.N) at week-6. A vaccine dose of 75 ugs/ plasmid DNA construct/animal, and 108 pfu/MVA construct/animal was administered at each immunization time point. Sera for ELISA and neutralizing antibody titers were harvested from blood collected at weeks-6 and -9. At week-6, three weeks post first boost, and at week-9, three weeks post final boost, mice (*n* = 5 each time-point) were euthanized, bronchoalveolar lavage (BAL) collected as described previously in D10 media [24], and lungs and spleen were harvested and processed for the intracellular cytokine staining (ICS) assay as described below.

### 2.4. SARS-CoV-2-Specific ELISA

Sera and BAL from different time-points were screened for humoral response against SARS-CoV-2 spike. To measure IgG and IgA antibody levels in the plasma and BAL of mice, respectively, a SARS-CoV-2-specific enzyme-linked immunosorbent assay (ELISA) was developed. Briefly, Nunc ELISA plates were coated with SARS-CoV-2 spike protein (BEI resources-NR-52396, 100 ng total/well) diluted in carbonate/bicarbonate buffer, pH 9.6, and incubated overnight at 4 °C followed by blocking with 5% skim milk to reduce background. A total of 100 μL of diluted serum (1/25) or BAL (undiluted) harvested at different time-points from immunized mice was added to the wells and incubated at 37 °C for 1 h. Post washing (PBS-TritonX 100, 0.1%), either HRP-conjugated anti-mouse IgG (1036-05, Southern Biotech, Birmingham, AL, USA) or anti-mouse IgA (1040-05, Southern Biotech) at dilutions of 1/1000 was added to the wells and incubated at 37 °C for 1 h. Post washing, 100 μL of TMB substrate solution was added and incubated for 20 min or until color developed. The addition of 1 M sulfuric acid stopped the reaction, and plates are read at 450 nm. Binding antibody end point titers (EPTs) were calculated as described previously [17].

### 2.5. SARS-CoV-2 Pseudovirus Neutralization Assay

SARS-CoV-2 pseudotyped virus based on HIV-pseudotyped the luciferase-reporter based system generated as described elsewhere was used to perform the neutralization assay [25]. The SARS-Related Coronavirus 2, Wuhan-Hu-1 Spike-Pseudotyped Lentiviral Kit, NR-52948, was obtained through BEI Resources, NIAID, NIH. For the neutralization assay, heat-inactivated sera were first serially diluted and incubated with the virus for 90 min at 37 °C. Then the serum–virus mixture was transferred into wells pre-seeded with HEK293T-hACE2 cells (BEI resources NR-52511). After 48 h, cells were lysed, and luciferase activity was measured using the ONE-Glo™ Luciferase Assay System (Promega, Madison, WI, USA). Relative light units (RLU) were measured using the TD 20/20 Luminometer (Turner Designs, San Jose, CA, USA). Neutralization titers (ID50) were calculated as the serum dilution at which RLU were reduced by 50% compared with RLU in virus control wells after subtraction of background RLU in cell control wells.

### 2.6. SARS-CoV-2 Wild-Type Virus Neutralization Assay

SARS-CoV-2, isolate USA-WA1/2020 obtained by Dr. Osorio from BEI resources, was propagated and titrated on Vero E6 cells. Human plasma samples were obtained through BEI Resources, NIAID, NIH: Human plasma, sample ID WU353-108, NR-53675-53679, contributed by Ali Ellebedy, Ph.D., Washington University School of Medicine, St. Louis, MO, USA. Heat-inactivated sera and BAL were first serially diluted in serum-free Opti-MEM media and incubated with 100 PFU per well of SARS-CoV-2, isolate USA-WA1/2020 for 60 min at 37 °C and transferred into wells pre-seeded with Vero E6 cells. Serum and BAL dilutions were performed in duplicate. Plates were incubated at 37 °C for four days before scoring for the cytopathic effect. The neutralization titer was calculated as the reciprocal of the highest dilution at which virus neutralization occurred.

### 2.7. Flow Cytometric Assessment of SARS-CoV-2-Specific Intracellular Cytokine Assay

Immunized C57BL/6 mice (*n* = 5) from each vaccine group 3 weeks post final boost were euthanized and used for flow cytometric assessment. Single-cell suspensions from the lungs and spleen were prepared using standard techniques. Briefly, lungs were excised and placed in a gentleMACS Dissociator M tube (Miltenyi 130-093-236) with 3 mL collagenase B (1 mg/mL, Roche). Lung tissue was processed using the gentleMACS Dissociator, followed by incubation for 30 min at 37 °C. Single-cell suspensions lung and spleen were prepared by gently squeezing through a 70-mm cell strainer (Falcon) after lysing red blood cells (RBCs) using 1X BD Biosciences BD Pharm Lyse™. For intracellular cytokine staining, 1 × 10^6^ cells were stimulated with SARS-CoV-2 spike protein (BEI resources-NR-52396, 100 ng total/well) overnight (~18 h) at 37 °C. Brefeldin A (1 μL/mL, GolgiPlug, BD Biosciences) was added after, and cells were further incubated for another 5 h at 37 °C. Fluorochrome-labeled antibodies against the cell-surface antigens CD4 (BUV 496, GK1.5), CD8a (BUV395, 53-6.7) and intracellular antigens IFN-γ (APC, XMG1.2), TNF-α (BV421, MP6-XT22), IL-2 (PE-CF594, JES6-5H4), IL-17 (FITC, TC11-18H10.1), IL-13 (PE-Cy7, eBio13A) were purchased from BD Biosciences (San Jose, CA, USA), Biolegend (San Diego, CA, USA), eBioscience (San Diego, CA, USA), or Invitrogen (Grand Island, NY, USA). Before antibody staining, cells were stained for viability with Dye eFluor 780 (eBiosciences, San Diego, CA, USA). After stimulation, cells were stained for surface markers and then processed with the Cytofix/Cytoperm Kit (BD Biosciences, NJ, USA). To stain for cytokines, cells were first stained for cell surface molecules, fixed, permeabilized, and subsequently stained for the cytokines. All samples were acquired on an LSR Fortessa (BD Biosciences) flow cytometer. Data were analyzed with FlowJo software (TreeStar, Woodburn, OR, USA). Results were expressed as the difference in the percentage of stimulated cells with that of unstimulated cells. At least 100,000 events were collected for each sample. A boolean gating strategy was applied for the determination of cytokine secreting T-cells (Figure S1). Individual type I cytokines expressed in lungs and spleen in response to antigen stimulation are highlighted in Figures S2 and S3.

### 2.8. Statistical Analysis

Statistical analyses were performed using GraphPad software (La Jolla, CA, USA). ELISA EPT and BAL nAb titers were compared using a Student’s *t*-test where *, *p* < 0.05; **, *p* < 0.01 were considered significantly different among groups. Serum neutralizing antibody titers and cellular immune assays were compared using an ordinary one-way ANOVA test where *, *p* < 0.05; **, *p* < 0.01 were considered significantly different among groups. Antibody titers (ELISA) were compared using a two-way ANOVA test where *, *p* < 0.05; **, *p* < 0.01 were considered significantly different.

## 3. Results

### 3.1. Design and Construction of SARS-CoV-2 Vaccine Constructs

**Figure 1:**
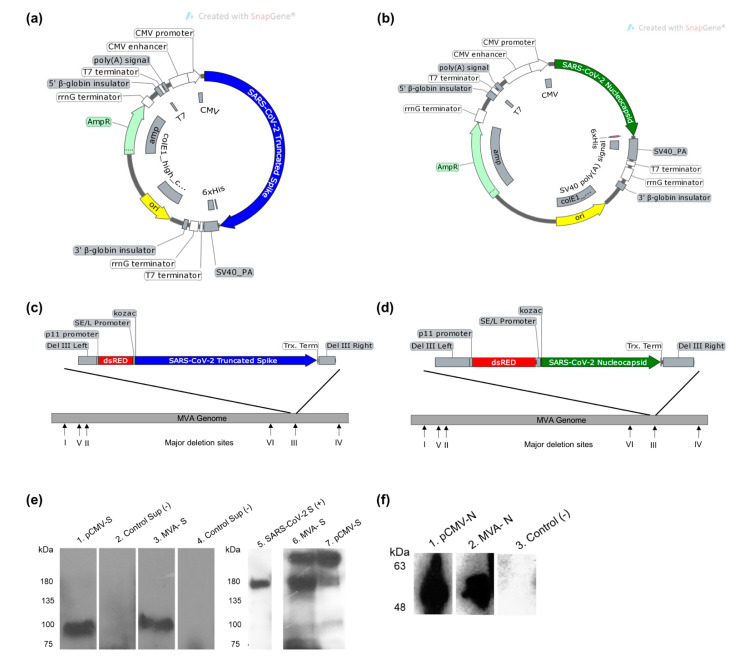
Design of SARS-CoV-2 vector vaccine constructs expressing S and N proteins. (**a**) Gene map of pCMV plasmid expressing Truncated S (TrS) protein. (**b**) Gene map of pCMV plasmid backbone expressing N protein. (**c**) MVA vaccine construct expressing TrS protein with the addition of C-terminal 6X His tag. (**d**) The MVA construct expressing N protein. All gene maps were generated using Snapgene software. (**e**) Western blot analysis with anti 6xHis-HRP antibody (left) and polyclonal mouse anti-SARS-CoV-2 spike sera (right) confirming expression of S protein from vaccine constructs. Lanes are as follows: Supernatant (lanes 2) HEK 293T cells transfected with control plasmid, supernatant (lanes 1 and 7) HEK 293T cells transfected with pCMV-TrS plasmid. Supernatant (lanes 3 and 6) from chicken embryonic fibroblast (CEF) cells infected with MVA-TrS and control non-infected supernatant (lane 4). Purified recombinant SARS-CoV-2 S Glycoprotein (BEI resources-NR-52396). (**f**) Western blot analysis with anti 6xHis-HRP antibody N from vaccine constructs. Lanes are as follows: Cell pellet (lane 3) HEK 293T cells transfected with control plasmid, cell pellet (lane 1) HEK 293T cells transfected with pCMV-N plasmid. Cell pellet (lane 2) from CEF cells infected with MVA-N.

Plasmid DNA (pDNA) vaccine candidates encoding the spike (S) gene of SARS-CoV-2 with a deletion of the transmembrane domain (TrS) and nucleocapsid gene (N) with the addition of C-terminal 6xHis tag independently were constructed. The genes from the first sequenced Wuhan SARS-CoV-2 isolate (GenBank accession number MN908947) were used for constructing the vaccine candidates. The expression of transgenes from the plasmid DNA vaccine candidates was confirmed using Western blot analysis on supernatant and cells harvested from transfected HEK-293T cells with an anti-6xHis antibody (Figure 1a,b). The same genes were cloned into the MVA shuttle vector that enables homologous recombination with and insertion into the deletion III region within the MVA genome (Figure 1c,d). The TrS and N protein expression in MVA vaccine candidates are under the control of the SE/L promoter. Western blot analysis confirmed protein expression in supernatant from pCMV-TrS and cell pellets from pCMV-N transfected 293T cells (Figure 1e,f and Figures S5 and S6). Similarly, protein expression in supernatant from MVA-TrS and cell pellets from MVA-N infected CEF cells (Figure 1e,f, and Figures S5 and S6) was confirmed. For TrS expression from both pDNA and MVA constructs, a single band of about 90 kDa was detected by the anti-6xHis antibody, reflecting the proteolytic cleavage of the secreted protein (Figure 1e). Three major bands, around 250, 180, and 90 kDa, were observed when mouse sera reactive to SARS-CoV-2 spike was used for Western blot analysis. These bands indicate the presence of both full-length and cleaved secreted spike proteins from the plasmid DNA and MVA vaccine candidates despite the presence of the furin cleavage site of the spike protein (Figure 1e).

### 3.2. Induction of SARS-CoV-2-Specific Humoral Responses in Vaccinated Mice

**Figure 2:**
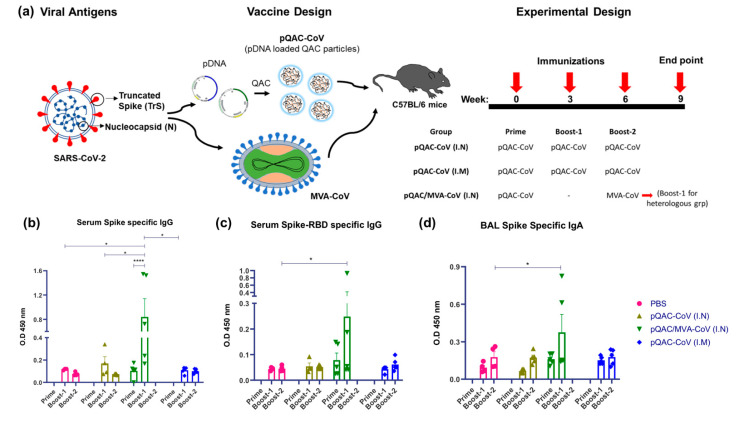
Generation of humoral immune responses in C57BL/6 mice following immunization with different vaccine constructs. (**a**) Outline for vaccine construct and immunization protocol using groups of C57BL/6 mice vaccinated with 3 doses of pQAC-CoV (I.N) or pQAC-CoV (I.M) with 3-week interval. Another group of C57BL/5 mice were vaccinated with pQAC-CoV (I.N) at week-0 followed by boost with MVA-CoV (I.N) at week-6. (**b**) ELISA titers of SARS-CoV-2 S-specific IgG in mice sera, (**c**) ELISA titers of SARS-CoV-2 spike receptor-binding domain (RBD)-specific IgG in mice serum and (**d**) ELISA titers of SARS-CoV-2 S-specific IgA in bronchoalveolar lavage (BAL), significance (*, *p* < 0.05, ****, *p* < 0.0001) was determined by two-way ANOVA. Data show mean ± SEM.

Previously, we reported the ability of QAC complexed avian coronavirus DNA vaccines to elicit robust T-cell responses without complementing humoral responses [11]. We hypothesized that a heterologous strategy of priming with plasmid encoded-antigens and boosting with MVA expressing the same antigens would overcome this limitation and elicit both robust T-cell and humoral immune responses [26,27]. For this purpose, groups of C57BL/6 mice were initially immunized with QAC complexed plasmid DNA (pCMV-TrS and pCMV-N, both termed pQAC-CoV) via I.N or I.M routes followed by two doses at 3 and 6 weeks post initial immunization (75 ugs plasmid DNA/dose/mouse), bringing the total doses to 3 times. Another group of C57BL/6 mice was also initially immunized with pQAC-CoV, followed by a boost with MVA-CoV at week-6 both via the I.N. route, bringing the total doses to 2 times. Interestingly, three weeks after a prime and one boost immunization, S-specific and S receptor-binding domain (RBD)-specific IgG levels were detected in the sera from the heterologous vaccine group (pQAC/MVA-CoV), significantly higher than the rest of experimental groups (Figure 2a,b). Antibody levels above the baseline for the PBS-immunized group were also recorded for the pQAC-CoV I.N and I.M groups, albeit non-significant (Figure 2a,b). A single-dose administration of pQAC-CoV (heterologous group, prime time-point) also elicited detectable S- and RBD-specific IgG levels higher than observed in the PBS group (Figure 2a,b).

Finally, we also examined mucosal secretory IgA (sIgA) produced in the lower respiratory tract that is critical in limiting infection of many respiratory pathogens, including coronaviruses at the primary site of infection [28,29,30]. The S-specific IgA (IgA) levels in the bronchoalveolar lavage (BAL) of immunized mice in the heterologous vaccine group were significantly higher than the PBS group (Figure 2c). However, we did not record significant induction of sIgA in any plasmid-immunized mice, even after prime and two boost doses. The presence of sIgA and circulating IgG in pQAC/MVA-CoV immunized mice underscores the potential of this mucosal vaccine strategy to elicit local and systemic humoral responses.

### 3.3. Heterologous Vaccine Strategy Elicits Neutralizing and Binding Antibody Responses

**Figure 3:**
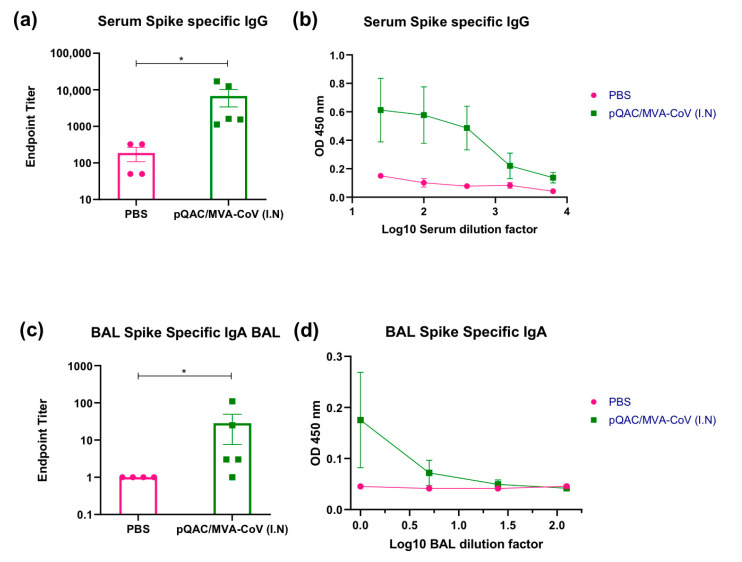
Heterologous vaccine strategy elicits spike-binding antibody responses. Groups of C57BL/5 mice were either unvaccinated (PBS) or immunized with pQAC/MVA-CoV (I.N). Serum and BAL samples were collected 3 weeks post boost. (**a**) SARS-CoV-2 spike-specific serum IgG binding endpoint titers measured by ELISA. (**b**) Serial serum dilutions of IgG binding to SARS-CoV-2 spike protein. (**c**) SARS-CoV-2 spike-specific BAL IgA binding endpoint titers measured by ELISA. (**d**) Serial BAL dilutions of IgA binding to SARS-CoV-2 spike protein. Significance (*, *p* < 0.05) was determined by one-way ANOVA. Data show mean ± SEM.

**Figure 4:**
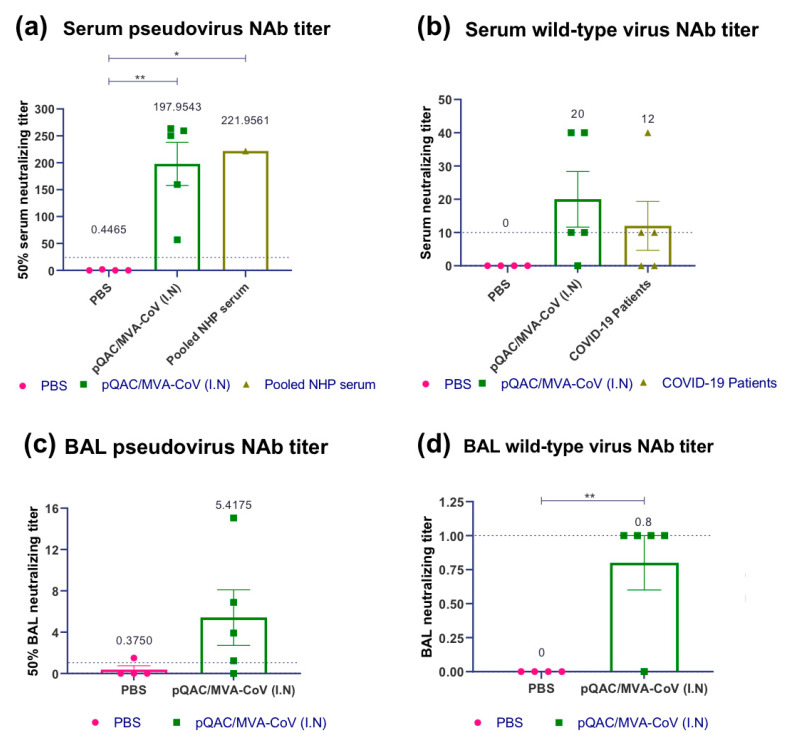
Heterologous vaccine strategy elicits SARS-CoV-2 neutralizing antibody responses. Groups of C57BL/5 mice were either unvaccinated (PBS) or immunized with pQAC/MVA-CoV (I.N). Serum and BAL samples were collected 3 weeks post boost. (**a**) 50% serum neutralization titer of pseudo-virus expressing SARS-CoV-2 spike, (**b**) serum neutralization titer of wild-type SARS-CoV-2, isolate USA-WA1/2020, (**c**) 50% BAL neutralization titer of pseudo-virus expressing SARS-CoV-2 spike, and (**d**) BAL neutralization titer of wild-type SARS-CoV-2, isolate USA-WA1/2020. Significance (*, *p* < 0.05; **, *p* < 0.01) was determined by one-way ANOVA. Data show mean ± SEM. The dotted line indicates the lower limit of detection.

To further characterize and accurately quantify humoral responses in the heterologous vaccine group (pQAC-CoV/MVA-CoV), we evaluated S-specific endpoint titers (EPT) using standard ELISA. Significant antibody titers ranging from ~1:1000–1:15,000 for circulating IgG (serum) and 1–1:100 for sIgA (BAL) were detected in the heterologous vaccine group (Figure 3a,c). To evaluate the neutralizing antibody response, we initially used a pseudovirus neutralization assay developed before using a recombinant lentivirus [25] expressing S protein from SARS-CoV-2. Unlike the PBS group, significant 50% neutralization titers ranging from ~1:50–1:250 (mean ~198) were observed in sera harvested from immunized mice three weeks after the final boost with MVA-CoV (Figure 4a). The neutralization titers were comparable to the pooled convalescent non-human primate (NHP) serum (BEI Resources-NR-52401) harvested from SARS-CoV-2 infected NHP, highlighting the ability of the heterologous vaccine strategy to elicit humoral responses to similar levels as seen during SARS-CoV-2 infection. Pseudovirus neutralization titers were also detected in BAL samples of immunized mice, which were higher than those detected from the PBS immunized group (Figure 4c). Finally, we evaluated the ability of harvested sera and BAL to neutralize wild-type SARS-CoV-2 virus. As observed with the pseudovirus neutralization assay, both serum and BAL samples from vaccinated mice could efficiently neutralize wild-type SARS-CoV-2 (Figure 4b,d). In fact, the neutralization titers observed with pQAC/MVA-CoV sera were higher than what was observed with COVID-19 patient plasma samples (Figure 4b). These results highlight the ability of the heterologous vaccine strategy to elicit potent SARS-CoV-2 neutralizing local and systemic antibody responses. EPT and nAb calculations were not performed with serum and BAL samples from other experimental groups and other time points since significant levels of IgG in serum and IgA in BAL were not observed (Figure 2b,d).

### 3.4. SARS-CoV-2-Specific Cellular Responses in Vaccinated Mice

Next, we evaluated the ability of the experimental vaccines to elicit local and systemic SARS-CoV-2 S- and N-specific cellular immune responses. Intracellular cytokine staining (ICS) was performed with lung cells and splenocytes harvested from vaccinated mice three weeks post final boost (i.e., P/B for pQAC-CoV/MVA-CoV and P/2XB for pQAC-CoV alone). For S-specific immune responses, cells were stimulated with purified recombinant spike glycoprotein (BEI resources-NR-52396) from SARS-CoV-2 for 24 h before staining. For N-specific immune responses, cells were stimulated with an N protein-peptide array from the SARS-CoV (BEI Resources-NR-2670) for 5 h before staining based on earlier protocols [31,32]. Increased lung cell death was observed in a few samples and accordingly were excluded from the analysis.

**Figure 5:**
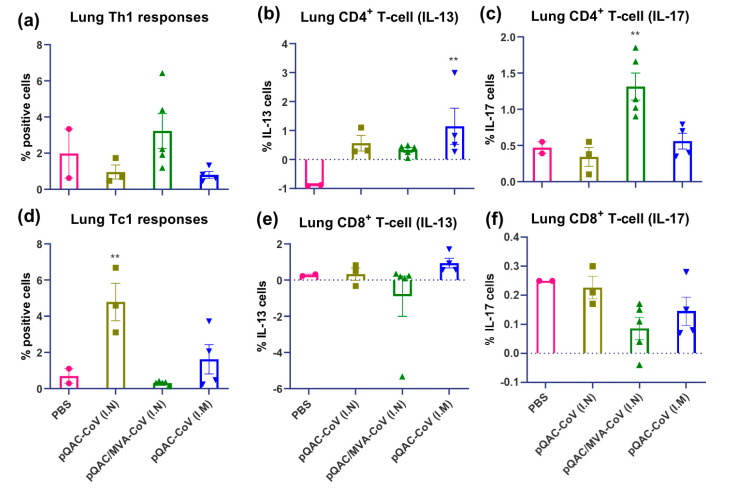
SARS-CoV-2 spike specific T cell responses in lungs of vaccinated C57BL/6 mice. Intracellular cytokine staining was performed on lungs harvested 3 weeks after final boost to assess T-cell responses. (**a**) Type 1 helper (Th1) responses (IFN-γ or TNFα or IL-2+), (**b**) type 2 helper (Th2) responses (IL-13+), (**c**) type 17 helper (Th17) responses (IL-17+), (**d**) type 1 cytotoxic (Tc1) responses (IFN-γ or TNFα or IL-2+), (**e**) type 2 cytotoxic (Tc2) responses (IL-13+), (**f**) type 17 cytotoxic (Tc17) responses (IL-17+) intracellular cytokine staining assays for lung T-cells in response to recombinant SARS-CoV-2 spike stimulation. Samples with lower live cells (<10,000) were excluded from analysis. Significance (**, *p* < 0.01) was determined by ANOVA compared to PBS controls. Data show mean ± SEM.

**Figure 6:**
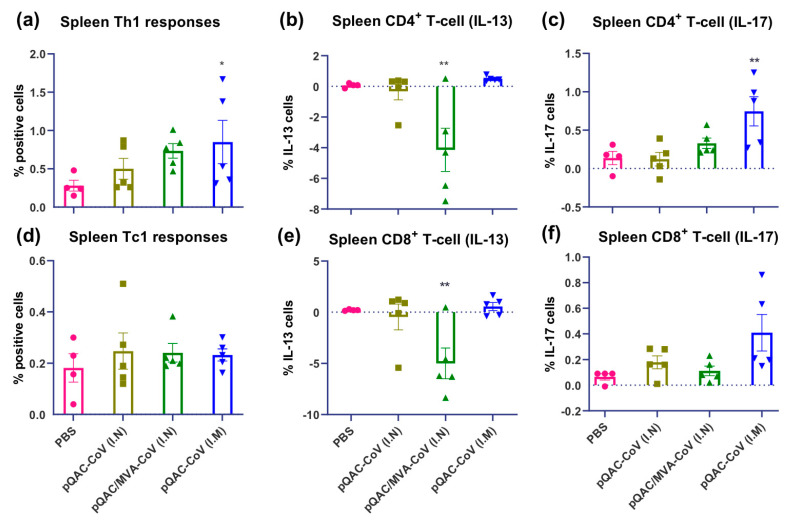
SARS-CoV-2 spike specific T cell responses in spleen of vaccinated C57BL/6 mice. Intracellular cytokine staining was performed on spleens harvested 3 weeks after final boost to assess T-cell responses. (**a**) Type 1 helper (Th1) responses (IFN-γ or TNFα or IL-2+), (**b**) type 2 helper (Th2) responses (IL-13+), (**c**) type 17 helper (Th17) responses (IL-17+), (**d**) type 1 cytotoxic (Tc1) responses (IFN-γ or TNFα or IL-2+), (**e**) type 2 cytotoxic (Tc2) responses (IL-13+), (**f**) type 17 cytotoxic (Tc17) responses (IL-17+) intracellular cytokine staining assays for spleen T-cells in response to recombinant SARS-CoV-2 spike stimulation. Significance (*, *p* < 0.05; **, *p* < 0.01) was determined by ANOVA compared to PBS controls. Data show mean ± SEM.

Interestingly, immunization with the heterologous vaccine induced significant S-specific Th17 (IL-17+) responses in the lungs (Figure 5). Th2 (IL-13+) and Tc2 responses were significantly lower in the heterologous vaccine group in the lungs and spleen of vaccinated mice (Figure 5 and Figure 6, respectively). Significantly higher S-specific Tc1 responses (predominantly IFN-γ+) were also present in the lungs of pQAC-CoV (I.N)-immunized mice without significant induction of CD4+ T-cells (Supplementary Figure S3), an observation noted previously with QAC-DNA vaccination in chickens [11]. In addition, intramuscular (I.M) administration of pQAC-CoV alone induced significantly higher Th1 and Th17 responses in the spleen of vaccinated mice (Figure 6). In one group, we sacrificed mice after a single-dose administration of pQAC-CoV, which revealed higher induction of TNFα + and IL2+ CD4+ T-cells in the lungs (data not shown), emphasizing the ability of pQAC-CoV to elicit early T-cell responses.

Moreover, bias towards Th1 cellular immune responses was observed (IL-2 and TNFα) after prime pQAC-CoV, which was then complemented by a Th17 response after MVA-CoV boost vaccination. T-cell immune responses against N were also evaluated but with no significant induction of CD4+ or CD8+ cells in both lungs and spleen. That being said, a statistically significant reduction in N-specific IL-13 producing CD8 and CD4 T-cells was observed in the lungs and spleen of all experimentally immunized mice (data not shown). These results highlight the ability of the heterologous vaccine strategy to elicit both potent local and systemic SARS-CoV-2 neutralizing antibody and cellular immune responses.

### 3.5. SARS-CoV-2 Vaccines Induce Polyfunctional T-Cells

**Figure 7:**
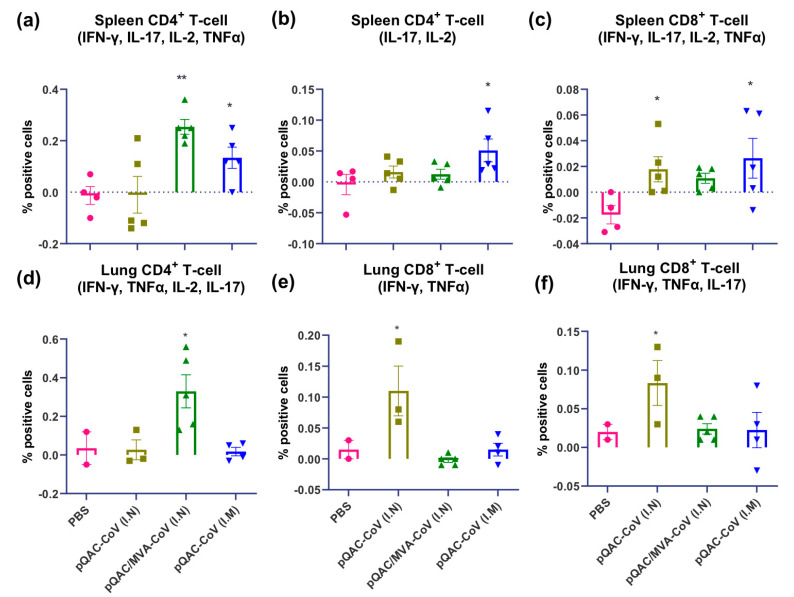
Immunization with SARS-CoV-2 vaccine constructs induces polyfunctional T cells. (**a**) CD4+ IFN-γ, TNFα, IL-2, and IL-17, (**b**) CD 4+ IL-2 and IL-17 (**c**) CD8+ IFN-γ, TNFα, IL-2, and IL-17 polyfunctional T-cells in the spleen of vaccinated mice. (**d**) CD4+ IFN-γ, TNFα, IL-2, and IL-17, (**e**) CD8+ IFN-γ and TNFα, (**f**) CD8+ IFN-γ, TNFα, and IL-17 polyfunctional T-cells in the lungs of vaccinated mice following intracellular cytokine staining assays for after recombinant SARS-CoV-2 spike stimulation. Significance (*, *p* < 0.05; **, *p* < 0.01) was determined by ANOVA compared to PBS controls. Data show mean ± SEM.

To further characterize cellular immune responses, we investigated the ability of the experimental vaccines to induce polyfunctional T-cells using ICS. Our analysis indicated a significant induction of polyfunctional CD4+ cytokine producing T-cells (IFN-γ, TNFα, IL-2, and Il-17) in the lung and spleen of mice vaccinated with the heterologous vaccine (Figure 7a,d). In addition, we detected the significant induction of polyfunctional CD4+ T-cells (IL-17, IL-2) and CD8+ T-cells (IFN-γ, TNFα, IL-2+, and Il-17) in the spleens of pQAC-CoV-vaccinated mice via the I.M route (Figure 7b,c). We also detected significant induction of polyfunctional CD8+ T-cells (IFN-γ, TNFα, Il-17 and IFN-γ, TNFα) in the lungs of pQAC-CoV vaccinated mice via the I.N route (Figure 7e,f). Overall, analysis of cellular immune responses indicated that pQAC-CoV-based vaccines administered via the I.N route induced significant local responses in the lung and those given via the I.M route in the spleen, as expected.

## 4. Discussion

Although many experimental vaccines against SARS-CoV-2 are currently in clinical trials and some approved, multiple vaccine approaches are needed to realistically cover immunizing large populations and achieving herd immunity [15,16,17,18]. As noted before, the upper respiratory mucosa is the primary site of SARS-CoV-2 infection, and local mucosal immune responses could potentially be critical in limiting SARS-CoV-2 dissemination into the lower respiratory tract and subsequent pneumonia [33]. Local mucosal immune responses such as airway epithelium T-cell responses and IgA humoral responses are critical for restricting respiratory viral pathogens like SARS, MERS CoV, etc. [28,29,34,35]. More recently, early immune responses against SARS-CoV-2 have been shown to be dominated by mucosal IgA [29]. The study concluded that mucosal IgA might be critical in limiting viral transmission and vaccines eliciting SARS-CoV-2 IgA could be more potent [29]. Mucosal vaccination strategies such as intranasal vaccinations could be an effective therapeutic strategy against COVID-19 by mediating the induction of local and systemic immune responses. Although appealing, formulating intranasal vaccinations can be challenging owing to the low bioavailability, antigen uptake, and antigen degradation at mucosal surfaces [36,37]. Previously, we detailed the development of the QAC adjuvant system for efficient intranasal delivery of DNA immunogens, circumventing some of the challenges observed with I.N vaccination [11]. The rational design specifically targeted the development of intranasal vaccine delivery route based on the known mucoadhesive properties of chitosan and the use of the potent immune-stimulant quil-A for combating mucosal pathogens such as SARS-CoV-2. In this study, we described the pre-clinical development of a mucosal, 2-dose heterologous vaccine candidate against COVID-19 using the QAC adjuvant system. We were able to rapidly develop and characterize plasmid DNA and MVA encoding SARS-CoV-2 spike (S) and nucleocapsid (N) constructs as soon as sequences were available on the GenBank database. The P/B with pQAC-CoV/MVA-CoV in mice led to the development of neutralizing antibody responses in serum (systemic) and BAL (local). Significant local and systemic T-cell responses against SARS-CoV-2 S were also observed in vaccinated mice. Moreover, no signs of respiratory distress and inappetence were noticed in vaccinated mice, highlighting the safety of the proposed vaccine approach.

Earlier studies have shown that heterologous DNA/virus immunization is more immunogenic and protective than homologous virus/virus immunization strategies [38]. Based upon our previous experience with the avian coronavirus [11] and other studies with SARS-CoV [39,40], we selected both nucleocapsid (N) and spike (S) proteins as our antigen targets. Although we immunized mice concurrently with both the N and S vaccine constructs, we focused on characterizing the more biologically relevant humoral responses against spike, which neutralized and limited viral entry [41,42]. Mice immunized with the pQAC/MVA-CoV vaccine candidate induced serum IgG and BAL IgA capable of binding to both the full-length spike and the RBD of the spike. Neutralizing antibodies (nAb) against SARS-CoV-2 have been shown to bind to the spike RBD and limit viral entry [42]. Likewise, serum and BAL harvested from vaccinated mice were able to efficiently neutralize the pseudovirus expressing SARS-CoV-2 spike, which was comparable to neutralization observed with convalescent serum from SARS-CoV-2-infected NHP. Independent studies have shown that convalescing NHP are protected against SARS-CoV-2 and SARS-CoV re-infection, highlighting the potential of neutralizing antibodies induced in pQAC/MVA-CoV-immunized mice to prevent SARS-CoV-2 infection [43,44]. Other studies have shown that pseudovirus neutralization correlates positively with infectious virus neutralization and might protect against the SARS-CoV-2 challenge [45]. Although we did not perform challenge studies, we did observe that sera and BAL harvested from pQAC/MVA-CoV-immunized mice were able to neutralize wild-type SARS-CoV-2 efficiently. Although we could not directly compare against currently approved COVID-19 vaccines, the heterologous vaccine strategy, like approved vaccines, has NAb titers comparable to or higher than seen with convalescent serum from COVID-19 patients.

Generally, vaccinia viral vectors (e.g., MVA) and chitosan adjuvants induced robust T-cell responses, including induction of pro-inflammatory cytokines like IFN-γ, TNFα, IL-2, Il-17, etc., which have been implicated in limiting a plethora of viral infections such as herpes simplex virus-1 and 2, West Nile virus, Simian Immunodeficiency Virus,, Respiratory Syncytial Virus, and influenza [6,46,47,48,49,50,51]. Although Tc17 cells, characterized by the secretion of IL-17 themselves, are not cytotoxic like IFN-g+ CD8+ T-cells, they can directly activate and prime cytotoxic CD8+ T-cells [48]. IL-17 ablation in RSV-infected mice led to increased airway inflammation and airway mucus, indicating a potential role for IL-17 in limiting inflammation [49]. IL-17 is critical for recruiting B cells to the lung in response to influenza infection by inducing CXCL13 expression [50]. IL-17 can also promote migration and differentiation of B1a cells, the primary source of IgM production [52]. Interestingly, pQAC/MVA-CoV-immunized mice have modest titers of IgA and IgG, and a significant percentage of Th17 cells reactive to SARS-CoV-2 S, which was not observed in other groups. This observation could suggest that immunization, coupled with IL-17 production, amplified SARS-CoV-2 S-specific humoral responses, a potential mechanism of action for the heterologous vaccine candidate. That being said, the presence of IL-17-producing CD4+ T-cells in the spleen of pQAC-CoV (I.M) did not correlate with antibody levels. Concurrent expression of pro-inflammatory cytokines is a hallmark of Tc17 and Th17 cells [53]. Similarly, we observed polyfunctional CD8+ T-cells and CD4+ T-cells in the lungs and spleen of pQAC/MVA-CoV-immunized mice. Although cellular responses were significant, the Th1 cytokine responses were not as robust as typically seen in C57BL/6 mice used in this study. The exact reason for a lack of a large amount of Th1 cytokine production is unclear. A possibility is that robust Th1 responses could have been generated earlier and the exact kinetics of vaccine-elicited T-cell responses need to be investigated.

As expected, we recognize that the presence of IL-17 among elicited cytokines could be a double-edged sword. For example, increased tissue pathology during viral infections has been observed, facilitated by promoting a Th2 response, including IL-13 production [53]. In our hands, we noticed no change in IL-13 producing cells in the lungs and spleen of pQAC/MVA-CoV-vaccinated mice compared to the control group. Negative stimulation seen with IL-13 producing cells most likely is baseline and is not biologically relevant. It potentially means that there was no stimulation and that the stimulation might have killed some cells. Previously, Th2 biased responses orchestrated by IL-13 were usually associated with vaccine-associated enhanced respiratory disease (VAERD) [15]. T-cell responses elicited against N protein were minimal, which could be explained by the fact that the SARS-CoV N peptide pool was used for stimulation initially when SARS-CoV-2 N peptide array was unavailable. However, reduction in IL-13 production was observed in the vaccinated mice highlighting the absence of Th2 responses. In an independent experiment we evaluated the ability of MVA-S single-dose vaccine to elicit SARS-CoV-2-specific immune responses. Immunization led to induction of ELISA detectable S-specific IgG in the serum (Figure S4a). Interestingly, there was no induction of S-specific IgA in the BAL and local Th17 immune responses in the lungs as seen with the pQAC/MVA-CoV vaccination (Figure S4b,c). This underscores the need for QAC-encapsulated DNA prime immunization prior to MVA immunization for induction of local and systemic immune responses. Nonetheless, the pQAC-CoV plasmids administered via the I.N route could induce potent S-specific Tc1, specifically CD8+ IFN-γ cells in the lung. Similarly, pQAC-CoV administered via the I.M route was also able to induce potent S-specific Th1 and Th17 cellular responses in the spleen. The roles of local and systemic IFN-γ producing T-cells in limiting coronaviruses and other respiratory viral pathogens have been reported previously in multiple reports [54]. This observation is consistent with our prior report on the QAC-complemented IBV vaccine where local T-cell immune responses were observed without induction of humoral responses after homologous plasmid immunization [11]. We focused on detailing the immune responses in the lower respiratory tract, which will be important in the context of preventing severe disease and hospitalizations. Mucosal responses in the upper respiratory can be critical in limiting the establishment of infection and subsequent disease. sIgA in nasal washes and identification of SARS-CoV-2-specific T-cells in the NALT and nasal mucosal region using ICS in response to vaccination can be the focus of future studies.

## 5. Conclusions

Overall, the work presented here profiles the type of immune responses elicited by different mucosal vaccination strategies. Based on presented results, we theorize that the heterologous vaccine strategy might be better at providing sterilizing immunity against SARS-CoV-2. The heterologous vaccination elicited both local and systemic humoral and T-cell immune responses. Moreover, SARS-CoV-2-specific IgA, like those elicited by the heterologous vaccine in this study, was shown to be more potent than IgG equivalents underscoring their importance in protecting against SARS-CoV-2 [55]. The DNA homologous vaccination strategy might not be able to prevent SARS-CoV-2 infection but could limit severe disease with SARS-CoV-2-specific CD8+ local polyfunctional T-cells. Our rationale vaccine design against COVID-19 is dependent on optimizing and unraveling vaccine-elicited immunity following mucosal immunization before further testing in a challenge experiment, which this study clearly lacks. The relevance of vaccine-induced humoral and cell-mediated immune responses can only be ascertained with a challenge study. Based on data presented here, both vaccine constructs and vaccination strategies employed were safe and immunogenic in mice. However, the immune interference effects of N antigen, if any, need further exploration. Our studies suggested no dampening of S-specific immune responses when N antigen was used in vaccine constructs. A similar outcome could be envisioned for the pQAC/MVA-CoV heterologous vaccination. Finally, the heterologous vaccine strategy enjoyed the advantages of mucosal immunization detailed before and could be an example of developing rapid vaccines against COVID-19 and other future respiratory infections.

## Data Availability

All data are available on request. Please contact: adel.talaat@wisc.edu.

## Supplementary Materials

The following are available online at https://www.mdpi.com/2076-393X/9/2/132/s1, Figure S1: Representative gating strategy for flow cytometry data, Figure S2: SARS-CoV-2 spike-specific type I responses in spleens of vaccinated C57BL/6 mice, Figure S3: SARS-CoV-2 spike-specific type I responses in lungs of vaccinated C57BL/6 mice, Figure S4 SARS-CoV-2 spike-specific immune responses in lungs of single-dose MVA-S vaccinated C57BL/6 mice, Figure S5: Expression of SARS-CoV-2 TrS from pDNA and MVA constructs, Figure S6: Expression of SARS-CoV-2 N from pDNA and MVA constructs.
