# Supplementary material for: Localized and Systemic Immune Responses against SARS-CoV-2 Following Mucosal Immunization
Source: Vaccines (Basel). 2021 Feb 6;9(2):132. doi: 10.3390/vaccines9020132 (PMC7914464; doi:10.3390/vaccines9020132)
Supplement: Supplementary file 1 [file vaccines-09-00132-s001.pdf]

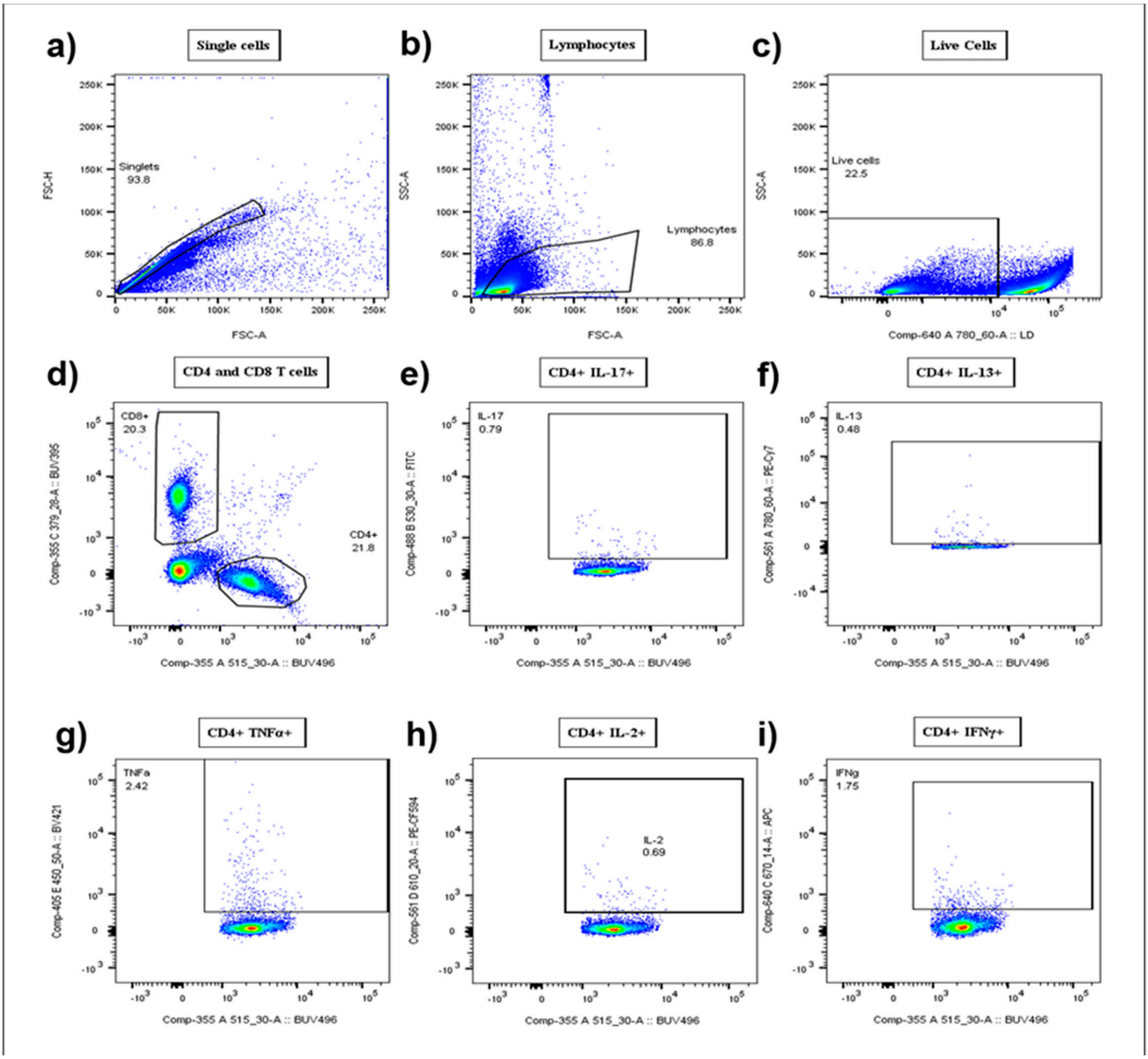

Figure S1. Representative gating strategy for flow cytometry data All samples (spleen and lung) had the following common gating: a) single cells, b) lymphocyte gate, c) live cells, d) CD4+ and CD8+ T-cells, e) IL17+ cells, f) IL-13+ cells, g) TNFα+ cells, h) IL-2+ cells and i) IFNγ+ cells.

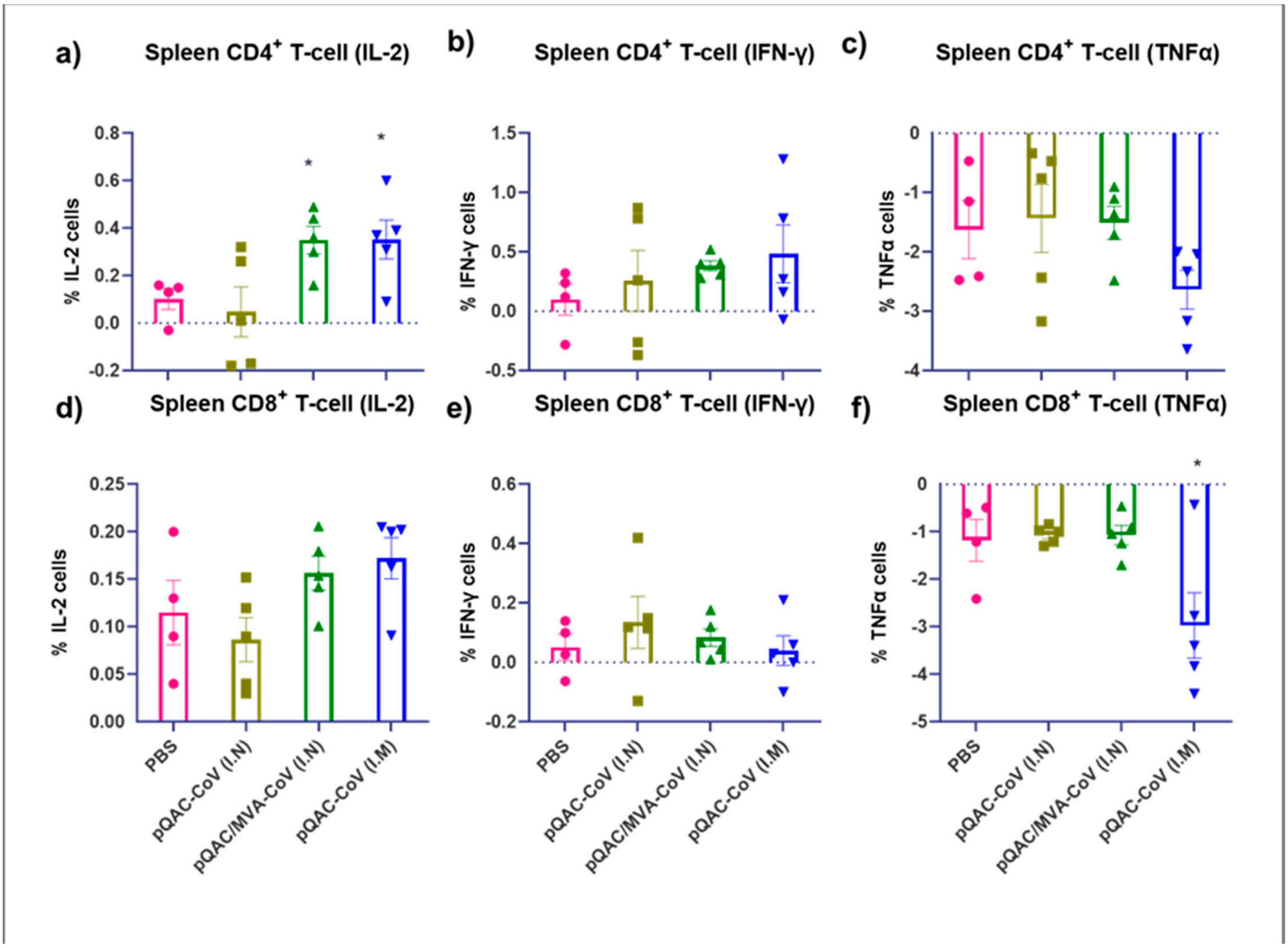

Figure S2. SARS-CoV-2 Spike specific type-I responses in Spleens of vaccinated C57BL/6 mice. Intracellular cytokine staining was performed on spleens harvested 3 weeks after final boost to assess T-cell responses. (a) IL-2+, (b) IFN-γ, (c) TNFα+ CD4+ T-cells and (d) IL-2+, (B) IFN-γ, (C) TNFα+ CD8+ spleen T-cells in response to recombinant SARS-CoV-2 Spike stimulation.

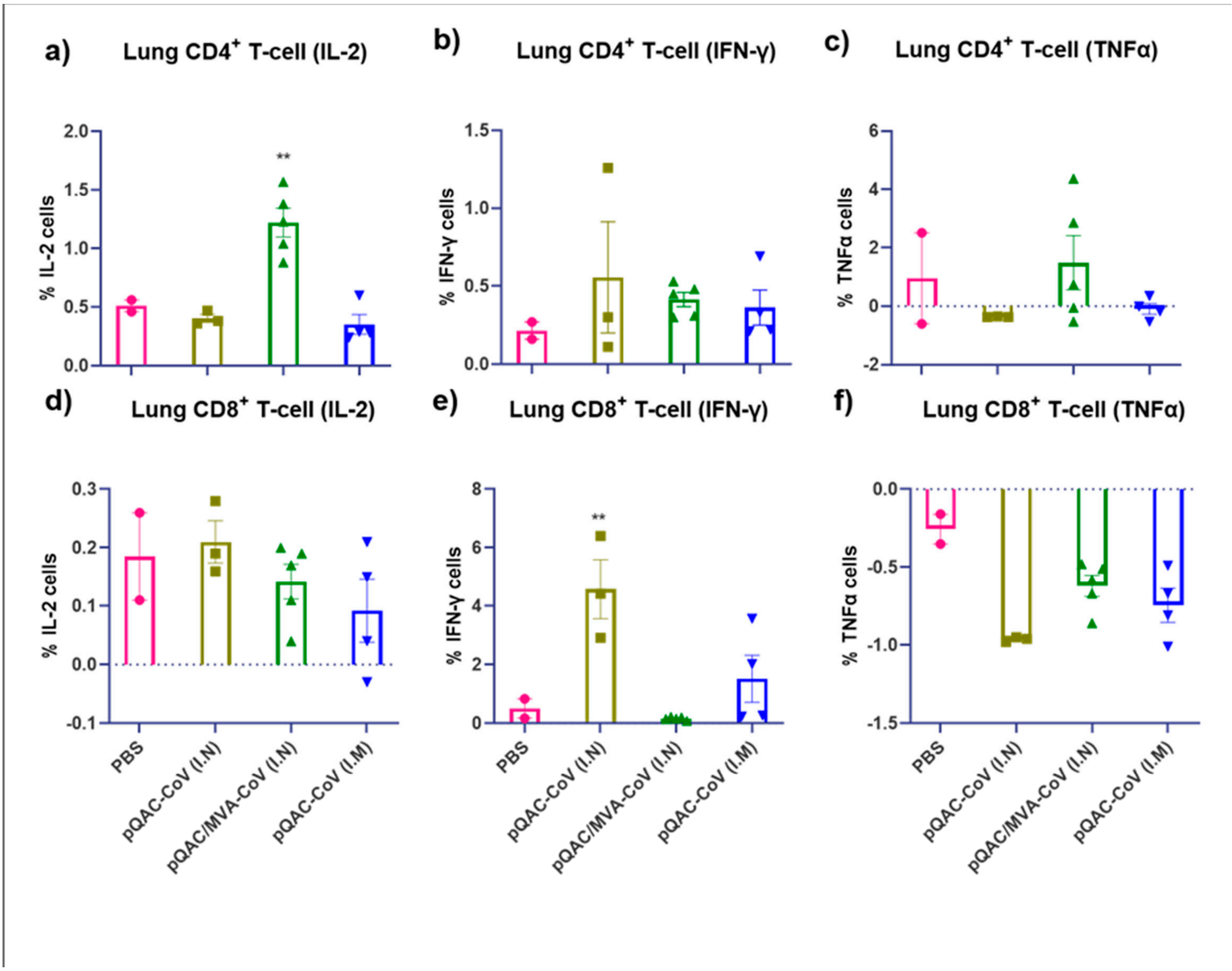

Figure S3. SARS-CoV-2 Spike specific type-I responses in Lungs of vaccinated C57BL/6 mice. Intracellular cytokine staining was performed on lungs harvested 3 weeks after final boost to assess T-cell responses. (a) IL-2+, (b) IFN-γ, (c) TNFα+ CD4+ T-cells and (d) IL-2+, (B) IFN-γ, (C) TNFα+ CD8+ lung T-cells in response to recombinant SARS-CoV-2 Spike stimulation.

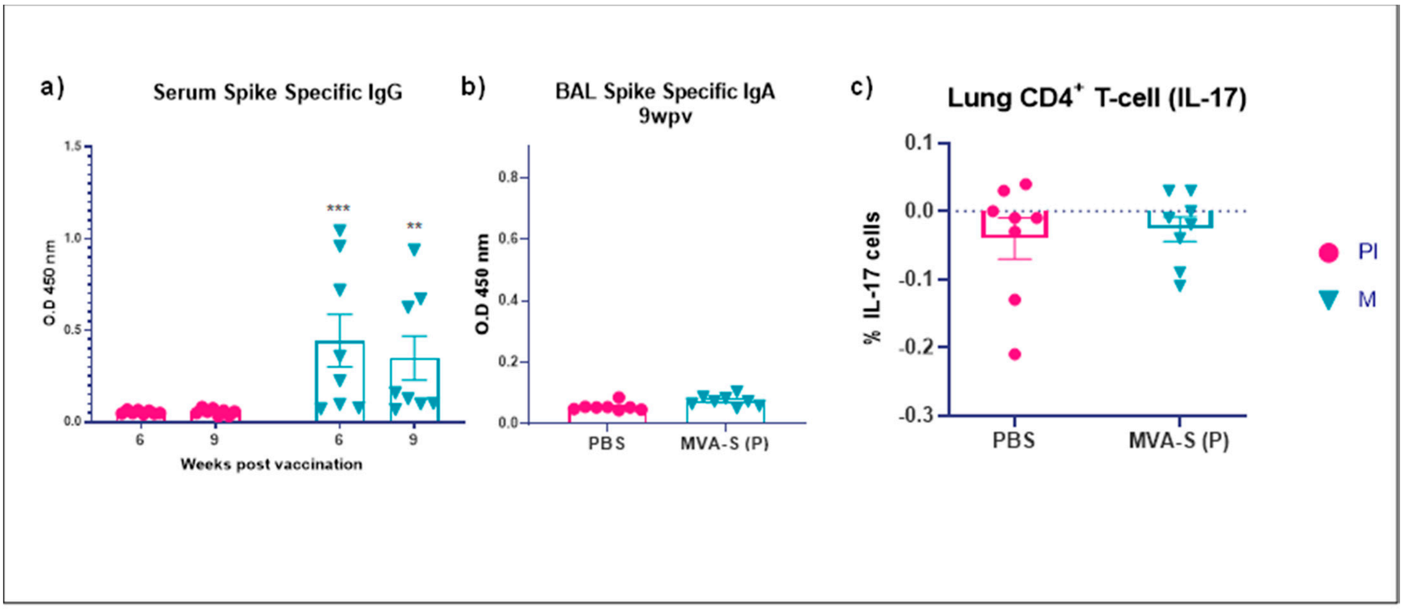

Figure S4. SARS-CoV-2 Spike specific immune responses in Lungs of single dose MVA-S vaccinated C57BL/6 mice. a) ELISA titers of SARS-CoV-2 S-specific IgG in mice sera, and b) ELISA titers of SARS-CoV-2 S-specific IgA in BAL, significance (**, P < 0.0021, ***, P<0.0002) was determined by two-way ANOVA. Intracellular cytokine staining was performed on lungs harvested 9 weeks after immunization to assess T-cell responses. (C) IL-17+ CD4+ lung T-cells in response to recombinant SARS-CoV-2 Spike stimulation.

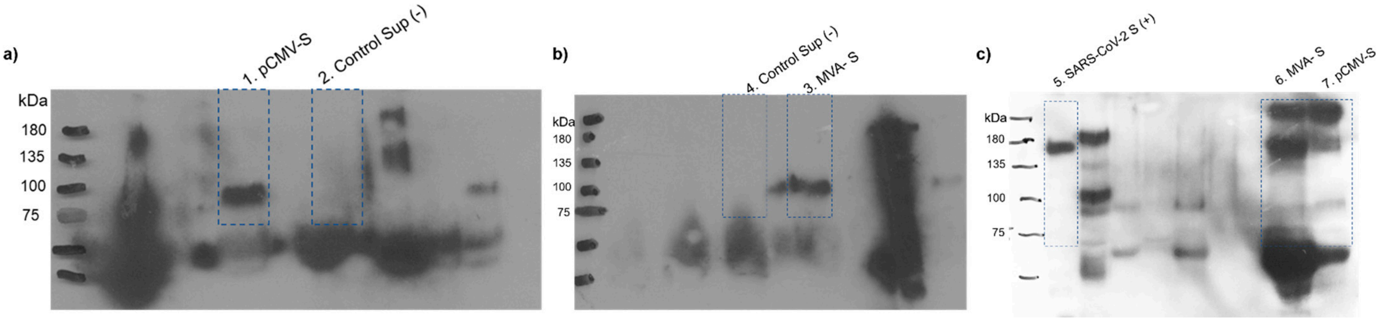

Figure S5. Expression of SARS-CoV-2 TrS from pDNA and MVA constructs. Full western blot images with anti 6xHis-HRP antibody (a and b) and polyclonal mouse anti-SARS-CoV-2 Spike sera (c) confirming expression of S protein from vaccine constructs. Lanes are as follows: Supernatant (lanes 2) HEK 293T cells transfected with control plasmid, Supernatant (lanes 1 and 7) HEK 293T cells transfected with pCMV-TrS plasmid. Supernatant (lanes 3 and 6) from CEF cells infected with MVA-TrS and control non-infected Supernatant (lane 4). Purified recombinant SARS-CoV-2 S Glycoprotein (BEI resources-NR-52396). The region of the western blot shown in Figure. 1e is boxed.

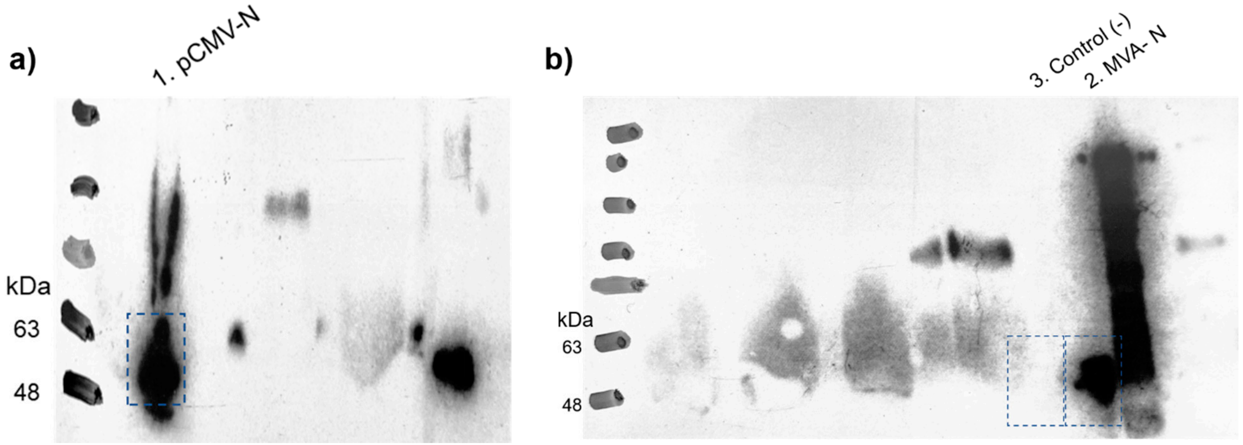

Figure S6. Expression of SARS-CoV-2 N from pDNA and MVA constructs. Full western blot images with anti 6xHis-HRP antibody confirming expression of S protein from vaccine constructs. Lanes are as follows. Cell pellet (lane 3) HEK 293T cells transfected with control plasmid, Cell pellet (lane 1) HEK 293T cells transfected with pCMV-N plasmid. Cell pellet (lane 2) from CEF cells infected with MVA-N.
